# Response of soil extracellular enzyme activity and stoichiometry to short-term warming and phosphorus addition in desert steppe

**DOI:** 10.7717/peerj.16227

**Published:** 2023-10-19

**Authors:** Lingxia Feng, Bing Cao, Xiaojia Wang

**Affiliations:** 1 School of Agriculture, Ningxia University, Yinchuan, China; 2 State Key Laboratory Cultivation Base for Northwest Degraded Ecosystem Recovery and Reconstruction, Yinchuan, China

**Keywords:** Soil available phosphorus, Extracellular enzyme activity, Phosphorus limitation, Desert steppe

## Abstract

**Background:**

Phosphorus (P) is regarded as one of the major limiting factors in grassland ecosystems. Soil available phosphorus deficiency could affect soil extracellular enzyme activity, which is essential for microbial metabolism. Yet it is still unclear how soil available phosphorus affects soil extracellular enzyme activity and microbial nutrient limitation of desert steppe in the context of climate warming.

**Methods:**

This study carried out a short-term open-top chambers (OTCs) experiment in a desert steppe to examine the effects of warming, P addition, and their interaction on soil properties, the activities of soil extracellular enzymes, and stoichiometries.

**Results:**

The findings demonstrated that soil acquisition enzyme stoichiometry of C: N: P was 1.2:1:1.5 in this experiment region, which deviated from the global mean scale (1:1:1). Warming increased soil AN (ammonium nitrogen and nitrate nitrogen) contents and decreased microbial biomass carbon (MBC) and microbial biomass nitrogen (MBN). Phosphorus addition raised soil available phosphorus and microbial biomass phosphorus (MBP) contents. Soil extracellular enzyme activities and stoichiometries in desert steppe are largely impacted by soil AN, MBC: MBP, and MBN: MBP. These results revealed that the changes of soil available nutrients and stoichiometries induced by short-term warming and P addition could influence soil microbial activities and alleviate soil microbial carbon and phosphorus limitation. Our findings highlight that soil available phosphorus played a critical role in regulating soil extracellular enzyme activity and microbial nutrient limitation of desert steppe. Further research on soil microbial communities should explore the microbiological mechanisms underlying these findings.

## Introduction

Phosphorus (P), one of the most significant mineral elements, is crucial for plant development, substance synthesis, and energy metabolism (Sharma et al., 2020), but there is a severe global deficiency of soil available P due to climate change, anthropogenic actions, land utilization patterns, and other factors (Hinsinger, 2001; Hou et al., 2020). P has evolved into a major limiting factor for plant growth in grassland ecosystems (Du et al., 2020), which may have negative effects on primary productivity and other ecological processes (Hou et al., 2020). Since 1850–1900, the temperature of the atmosphere has risen by around 1.1 °C, and it is predicted to rise by another 1.5–2.0 °C or more in the future (IPCC et al., 2021). Climate warming may alter the availability of soil nutrients (Feike et al., 2012; Hu et al., 2022), and soil microbes secrete extracellular enzymes in response to the variation of soil nutrients (Raiesi & Salek-Gilani, 2018; Cui et al., 2019a). Soil extracellular enzymes mediate the decomposition of soil organic matter and nutrient cycling, affecting the function of ecosystem services (Güsewell, 2004; Hu et al., 2016). Soil extracellular enzyme secretion is frequently the rate-limiting step of microbial metabolism (Nannipieri, Trasar-Cepeda & Dick, 2018). In this process, temperature affects soil microbial activity (Zi et al., 2018). Global warming has the potential to change soil available phosphorus by altering mean annual temperature and precipitation (Hou et al., 2018), and may also impact phosphorus fixation, mineralization, and weathering by indirectly affecting plant growth and soil microbial community structure (Delgado-Baquerizo et al., 2013). Soil enzyme stoichiometry can be used to evaluate the degree of nutrient availability and limitation of the microbial environment (Moorhead et al., 2013; Sinsabaugh et al., 2008).

Climate change could increase the availability of nutrients by accelerating the microbial breakdown of soil organic matter (Bardgett, Freeman & Ostle, 2008). The demand for carbon and phosphorus from microbes may change as a result of short-term warming, leading to greater microbial phosphorus limitation (Zheng et al., 2020). P addition enhances the soil available phosphorus, reduces phosphorus limitation, and increases carbon sequestration (Manzoni et al., 2012; Wang et al., 2022). P addition has both enhancing and inhibitory impacts on soil extracellular enzyme activity (Jing et al., 2016; Wang et al., 2020a), and different chemical forms of phosphorus inputs have an impact on soil acquisition enzyme activities (Waring, Weintraub & Sinsabaugh, 2014; Wang et al., 2020b). These differing results indicate that the potential mechanisms influencing soil extracellular enzyme activity and microbial metabolism induced by soil available P in the context of climate change are still unknown.

Desert steppe as a typical fragile grassland ecosystem that is especially vulnerable to climate change (Zuo et al., 2020), and is important to sustaining ecological security and socioeconomic development (Kang et al., 2007). The degradation of desert steppe and the decline of ecosystem stability and service functions may be driven by climate change, overgrazing, and exploitation (Reynolds et al., 2007; Deng, Sweeney & Shangguan, 2014; Zhang, Su & Yang, 2019). Therefore, addressing the relationship between soil microbial nutrient limitation and available phosphorus in this study area could provide key insights into soil phosphorus cycling and other ecosystem processes as a result of climate warming (Wang et al., 2021). We set up a field experiment to investigate the response of soil extracellular enzyme activity and its stoichiometry to warming and P addition in desert steppe. This study attempts to answer the following two questions: (i) how are soil extracellular enzyme activity and microbial nutrient limitation impacted by short-term warming and P addition? and (ii) what are the major factors affecting soil extracellular enzyme activity and stoichiometry? We hypothesized that (i) the degree of nutrient limitation of soil microorganisms was closely related to the content of soil available phosphorus, and (ii) warming and P addition would significantly reduce soil extracellular enzyme activity in the desert steppe.

## Materials & Methods

### Study site

The experiment was conducted on a desert steppe in Eastern Yanchi County (37°04′–38°10′N, 106°03′–107°04′E), Ningxia Hui Autonomous Region, Northwest China. It is located on the southwest edge of the Mu Us Desert, which has a semiarid continental monsoon climate. According to meteorological data from the Yanchi Meteorological Station, the average annual temperature is 8.8 °C, and the average annual precipitation is 298.15 mm, with the majority of the precipitation falling between July and September (1980–2021). The soil type is classified as Arenosol (IUSS, 2015), with 4.12 g kg^−1^ of soil organic matter, 0.40 g kg^−1^ of total nitrogen, 0.32 g kg^−1^ of total P, 2.30 mg kg^−1^ of available P, and pH of 8.57. The dominant species in the region are *Agropyron mogolicum*, *Lespedeza potaninii*, *Caragana korshinskii*, *Stipa bungeana*, and *Polygala tenuifolia Willd*.

### Experimental design

We conducted a randomized split-plot design with two temperature treatments (Control, CK; Warming, W) as the main plot and three P addition levels (0 g m^−2^ yr^−1^, 5 g m^−2^ yr^−1^, and 10 g m^−2^ yr^−1^) as the subplot in April 2022. There were six treatments: P_0_, P_5_, P_10_, WP_0_, WP_5_, and WP_10_, each replicated four times, for a total of 24 treatment plots. A PVC sheet was put into the soil at a depth of 0.8 m to divide each subplot from the main plot, spaced 3 m apart to provide a buffer zone (Fig. S1). Phosphorus fertilizer was supplied by triple superphosphate (Ca (H_2_PO_4_)_2_ H_2_O), which was evenly distributed in the treatment plots before rainfall from early June to August. Although the rate of phosphorus fertilizer input in this study is higher than the rate at which phosphorus is deposited in the atmosphere of northern China (Zhu et al., 2016), it corresponds to the current agricultural fertilization level in China (Cui et al., 2020; Guo et al., 2022).

The experimental warming device was modified based on the meteorological data from the study site from 1980 to 2021 (Fig. S2) as well as previous research (Ma et al., 2019; Ma et al., 2021). Open top chambers (OTCs) were used to passively increase the temperature in this investigation. Stainless steel and high-transmittance glass material (5 mm thick) were used to construct a regular octagonal prism structure, with a substrate area of 5.6 m^2^ and a vertical height of 1.8 m. These were permanently installed in the sample plot to avoid disruption. The air and soil temperatures at 15 cm above and below ground were automatically recorded every half-hour using HOBO MX2302A and HOBO MX2201 (Onset Computer Corporation, Bourne, MA, USA) data loggers, respectively.

### Soil sampling and measurements

We sampled soils on August 25, 2022. Three soil columns (5 cm in diameter) were collected randomly from the surface soil (0–15 cm in depth) in each subplot. The soil samples were mixed after removing gravel and litter, and then homogenized and sieved through a 2-mm mesh sieve. The composite soil samples were then divided into two parts, the first subsample was air-dried to measure soil organic carbon (SOC), total nitrogen (TN), total phosphorus (TP), and available phosphorus (AP). The second subsample was stored at 4 °C to measure dissolved organic carbon (DOC), ammonium nitrogen (NH_4_^+^–N), nitrate nitrogen (NO_3_^−^–N), microbial biomass (MBC, MBN and MBP), and soil extracellular enzyme activities.

Soil moisture content (SMC) was determined by oven-drying the soil at 105 °C for 48 h. A PHS-3E glass pH electrode (Leici, Shanghai, China) was used to measure the pH of the soil in a suspension of air-dried soil and distilled water (1:5, w/v). Soil organic carbon (SOC) and total nitrogen (TN) were determined using the potassium dichromate external heating method and H_2_SO_4_-H_2_O_2_ digestion with the Kjeldahl method. Total phosphorus (TP) was determined by HClO_4_-H_2_SO_4_ digestion with the molybdenum antimony anti-colorimetric method (Nelson & Sommers, 1996). A TOC analyzer was used to measure dissolved organic carbon (Vario TOC, Elementar, Hanau, Germany). AN (NH_4_^+^–N and NO_3_^−^–N) was determined using the KCl extraction method with a continuous flow analyzer (AutoAnalyzer3, Bran Luebbe, Germany). Available phosphorus (AP) was determined by NaHCO_3_ extraction using the molybdenum antimony anti-colorimetric method; and microbial biomass (MBC, MBN, MBP) was extracted and analyzed by chloroform fumigation (Vance, Brookes & Jenkinson, 1987).

### Soil extracellular enzyme extraction and vector analysis

Soil C- (*β*-1,4-glucosidase, BG), N-(leucine aminopeptidase, LAP, and *β*-1,4-N-acetylglucosa minidase, NAG), and P- acquiring enzyme (alkaline phosphatase, ALP) activities were determined using a modified standard fluorescence technique (Saiya-Cork, Sinsabaugh & Zak, 2002; German et al., 2011). Soil extracellular enzyme activity (EEA) was expressed in units of nmol g^−1^ h^−1^ (Sinsabaugh et al., 2008; Sinsabaugh, Hill & Shah, 2009; Waring, Weintraub & Sinsabaugh, 2014). The ratios of C, N, and P acquisition enzymes were calculated using the following formulae to determine soil extracellular enzyme stoichiometry (EES), respectively:

Soil e C: N = ln (BG) /ln (LAP + NAG)

Soil e C: P = ln (BG) /ln (ALP)

Soil e N: P = ln (LAP + NAG) /ln (ALP)

Vector analysis of soil extracellular enzyme activity was used to identify microbial nutrient limitations (Hill et al., 2012; Jiang, Zhu & Zeng, 2022). Vector length and vector angle were calculated by the following formulae, respectively (Moorhead et al., 2013; Moorhead et al., 2016), with limitations on N and P shown by vector angles of less than 45° and greater than 45°, respectively, and greater C limitation is shown by a relatively longer vector length:

Relative C limitation = Vector Length = $\sqrt{[\ln (\mathrm{BG})/\ln (\mathrm{LAP}+\mathrm{NAG})]^{2}+[\ln (\mathrm{BG})/\ln (\mathrm{ALP})]^{2}}$

N/P limitation =Vector angle(°) = Degrees (ATAN2((ln(BG)/ln(ALP), (ln(BG)/ln(LAP+NAG)))

### Statistical analyses

A two-way analysis of variance using a split-plot design was carried out to investigate the effects of warming, P addition, and their interaction on soil extracellular enzyme activities and stoichiometries in desert steppe. One-way ANOVA was used to compare the differences in soil properties, soil extracellular enzyme activities, and stoichiometries of the different treatments. Significant differences were tested using Duncan’s multiple comparison tests (*P* < 0.05), and the data of soil enzyme activity was transformed using a natural logarithm before statistical analysis. Before performing an ANOVA, all data were tested for normality of the residuals and homogeneity of variances in SPSS 25.0 for Windows (SPSS Inc., Chicago, Illinois, USA). Pearson correlation analysis was used to determine the association between soil properties, microbial biomass, and stoichiometries. Mantel test was used to explore the correlation between C, N, and P acquisition enzyme activities and soil environmental factors. Redundancy analysis (RDA) was used to explore the corresponding relationships of soil properties, microbial biomass, and extracellular enzyme activity in Canoco 5.0. For the graphics, R 4.2.3 and Origin 2022 (Origin Laboratory Corporation, Northampton, MA, USA) were applied.

## Results

### Soil temperature and soil moisture content

**Figure 1:**
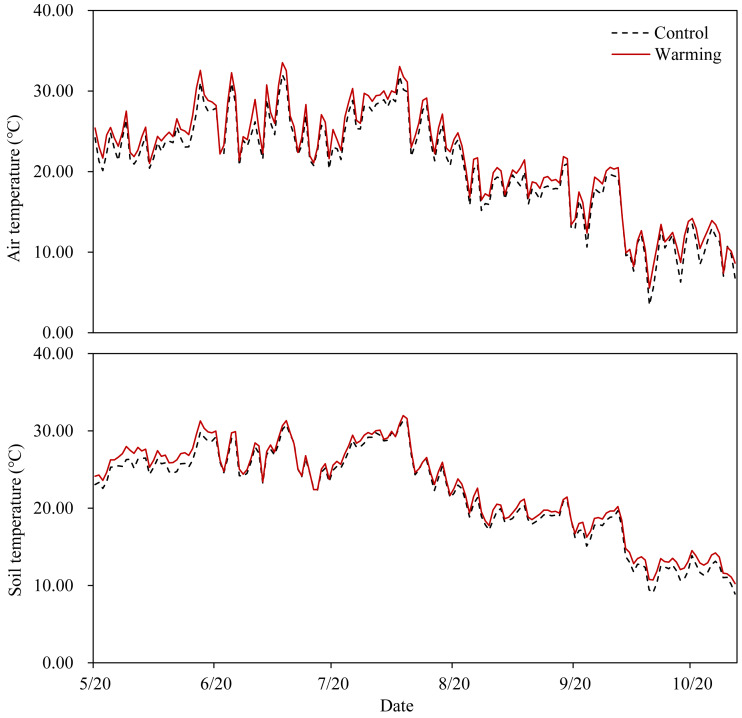
Air temperature at 15 cm above the ground and soil temperature at 15 cm below the ground in the warming plots (inside of the OTCs) and the control plots (outside of the OTCs).

OTCs had the anticipated warming effect in all temperature-increased treatment plots (Fig. 1), with an average increase of 1.15 °C in the air temperature 15 cm aboveground and an average increase of 0.65 °C in the soil temperature 15 cm belowground. Warming significantly decreased soil moisture content (SMC) by 13.67%, while P addition significantly increased SMC by 11.20% and 19.52% at P_5_ and P_10_, respectively. Soil moisture content was not significantly affected by warming × P addition (Table 1).

**Table 1:** Warming, P addition, and their interaction on soil properties, nutrients contents and stoichiometries.

Treatment		pH	SMC	SOC	TN	TP	SOC:TN	SOC:TP	TN:TP	DOC	AN	AP	DOC:AN	DOC:AP	AN:AP
Control	P_0_	8.55 ± 0.11a	9.73 ± 0.41b	6.98 ± 1.39ab	0.67 ± 0.12ab	0.34 ± 0.02c	10.46 ± 0.62a	20.27 ± 3.45a	1.94 ± 0.33a	20.79 ± 1.50abc	6.75 ± 0.52cd	3.58 ± 0.39d	3.09 ± 0.21a	5.83 ± 0.26a	1.9 ± 0.17a
	P_5_	8.53 ± 0.09ab	10.82 ± 0.93a	6.42 ± 0.79b	0.55 ± 0.12b	0.4 ± 0.04abc	11.92 ± 0.98a	16.18 ± 0.51bc	1.37 ± 0.16bc	17.77 ± 1.27c	6.30 ± 0.56d	30.43 ± 1.89b	2.83 ± 0.26a	0.59 ± 0.07c	0.21 ± 0.02c
	P_10_	8.48 ± 0.11ab	11.63 ± 1.20a	7.15 ± 1.34ab	0.62 ± 0.1ab	0.48 ± 0.09a	11.47 ± 0.58a	14.87 ± 2.19c	1.29 ± 0.13c	18.38 ± 2.19c	6.73 ± 0.34cd	41.60 ± 2.80a	2.74 ± 0.36a	0.44 ± 0.07d	0.16 ± 0.01c
Warming	P_0_	8.39 ± 0.05b	8.40 ± 0.26c	7.05 ± 0.99ab	0.66 ± 0.13ab	0.37 ± 0.08bc	10.77 ± 0.68a	19.3 ± 3.27ab	1.8 ± 0.36ab	19.75 ± 3.47bc	7.61 ± 1.40bc	3.80 ± 0.48d	2.62 ± 0.34a	5.21 ± 0.7a	1.99 ± 0.15a
	P_5_	8.44 ± 0.05ab	9.43 ± 0.46b	8.34 ± 0.9a	0.76 ± 0.13a	0.41 ± 0.07abc	11.11 ± 1.35a	20.56 ± 2.33a	1.89 ± 0.43a	23.33 ± 2.64ab	11.26 ± 0.78a	19.73 ± 3.15c	2.07 ± 0.11b	1.2 ± 0.21b	0.58 ± 0.07b
	P_10_	8.53 ± 0.11ab	9.59 ± 0.22b	7.33 ± 0.41ab	0.64 ± 0.06ab	0.47 ± 0.05ab	11.48 ± 0.88a	15.69 ± 2.06bc	1.37 ± 0.17bc	23.92 ± 2.66a	8.19 ± 0.82b	46.90 ± 6.78a	2.95 ± 0.52a	0.52 ± 0.12cd	0.18 ± 0.03c
Warming		—	*	—	—	—	—	—	**	—	**	—	—	*	*
P addition		—	**	—	—	**	—	*	*	—	*	***	*	***	***
Warming × P		—	—	—	—	—	—	—	—	*	**	***	**	***	*

**Notes.**

SMC soil moisture content SOC soil organic carbon TN soil total nitrogen TP soil total phosphorus DOC dissolved organic carbon AN soil ammonium nitrogen (NH_4_^+^–N) and nitrate nitrogen (NO_3_^−^–N) AP soil available phosphorus

The results of two-way split-plot ANOVAs for the treatments are shown in the table (^∗^*P* < 0.05,^∗∗^*p* < 0.01,^∗∗∗^*p* < 0.001, and “—” indicate no significance). Lowercase letters show significant differences. Among the treatments by Duncan’s test at *p* < 0.05.

### Soil properties

Warming had no significant effects on SOC, TN, and TP (Table 1). P addition did not affect SOC, but significantly increased TP and decreased TN. Warming × P addition increased SOC and TP. WP_10_ decreased TN by 4.48%, while WP_5_ increased it by 13.43%. Warming and P addition did not significantly affect SOC: TN, but decreased SOC: TP. Warming × P addition increased SOC: TP by 1.43% at WP_5_ and decreased by 22.59% at WP_10_. TN: TP decreased by 2.58% and 29.38% at WP_5_ and WP_10_, respectively.

Soil pH was not significantly affected by warming, P addition, and their interaction (Table 1). Warming and P addition both individually decreased DOC, but their interaction significantly increased DOC by 12.22% and 15.06% at WP_5_ and WP_10_, respectively. Warming and warming × P addition significantly increased AN. P addition decreased AN by 6.67% at P_5_ and did not change at P_10_. P addition had a considerable impact on available P, and the higher the rate of P addition, the greater the available P content. However, warming had no discernible influence on available P. Warming and P addition decreased DOC: AN. Warming × P addition decreased DOC: AN by 33.05% at WP_5_ and no significant effect at WP_10_. P addition and warming × P addition had a significant negative impact on DOC: AP and AN: AP.

### Soil microbial biomass and their stoichiometry

**Figure 2:**
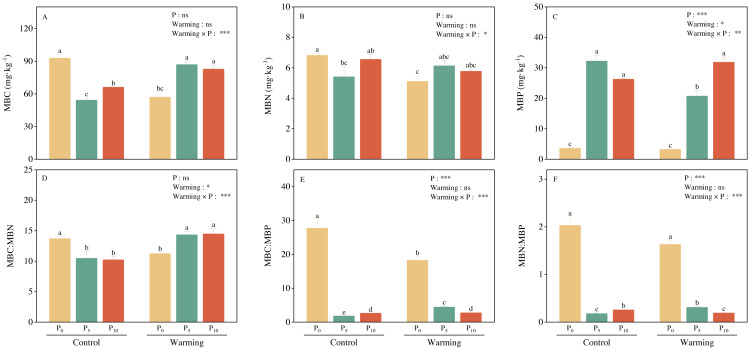
Warming, P addition, and their interaction on soil microbial biomass and stoichiometries. MBC, microbial biomass carbon; MBN, microbial biomass nitrogen; MBP, microbial biomass phosphorus. The results of two-way split-plot ANOVAs for the treatments are shown in the figures (*P < 0.05, ** *p* < 0.01, *** *p* < 0.001, and ns indicate no significance). Lowercase letters above the multi-factor group histograms show significant differences among the treatments by Duncan’s test at *p* < 0.05. Error bars show means ± SE (*n* = 4).

Warming and P addition did not significantly affect MBC and MBN, but their interaction significantly decreased both MBC and MBN (Figs. 2A and 2B). Warming reduced MBP by 9.01%. P addition and warming × P addition considerably increased MBP (Fig. 2C). Warming and P addition, individually, significantly decreased MBC: MBN, MBC: MBP, and MBN: MBP. Warming × P addition increased MBC: MBN (Fig. 2D), and substantially decreased MBC: MBP (Fig. 2E) and MBN: MBP (Fig. 2F).

### Soil enzyme activity and their stoichiometry

**Figure 3:**
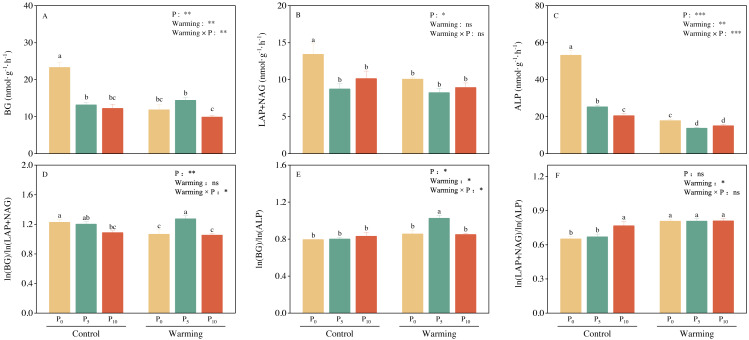
Warming, P addition, and their interaction on soil extracellular enzyme activities and stoichiometries. BG, *β*-1,4-glucosidase; LAP, leucine aminopeptidase; NAG, *β*-1,4-N-acetylglucosaminidase; ALP, alkaline phosphatase. The results of two-way split-plot ANOVAs for the treatments are shown in the figures (* *P* < 0.05, ** *p* < 0.01, *** *p* < 0.001, and ns indicate no significance). Lowercase letters above the multi-factor group histograms show significant differences among the treatments by Duncan’s test at *p* < 0.05. Error bars show means ± SE (*n* = 4).

**Figure 4:**
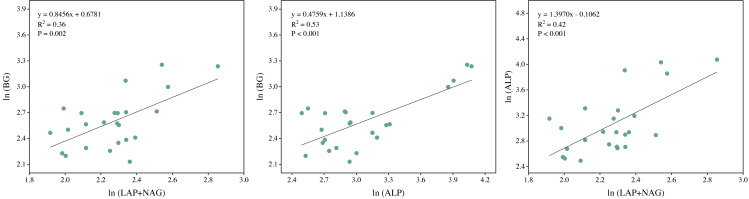
Regression analysis of the soil C, N, and P acquisition enzyme activities. BG, *β*-1,4-glucosidase; LAP, leucine aminopeptidase; NAG, *β*-1,4-N-acetylglucosaminidase; ALP, alkaline phosphatase. All data were natural log(ln)-transformed.

Soil BG (C-acquiring enzyme) was significantly decreased by warming, P addition, and their interaction (Fig. 3A). As for warming × P addition, BG at WP_5_ was higher than at WP_10_. LAP+NAG (N-acquiring enzyme) was significantly decreased by P addition (Fig. 3B), but not significantly affected by warming or warming × P addition. ALP (P-acquiring enzyme) was significantly reduced by warming, P addition, and warming × P addition (Fig. 3C). Warming and P addition reduced ln (BG): ln (LAP+NAG) (Fig. 3D), while warming × P addition increased ln (BG): ln (LAP+NAG) by 4.10% at WP_5_, and decreased it by 13.93% at WP_10_. Warming × P addition increased ln (BG): ln (ALP) at WP_5_ (Fig. 3E), but there were no significant changes observed on ln (BG): ln (ALP) from other treatments. Warming, P addition, and their interaction raised ln (LAP+NAG): ln (ALP) (Fig. 3F). A positive correlation between soil C-, N-, and P-acquiring activity was also discovered (Fig.  4).

### Vector analysis

**Figure 5:**
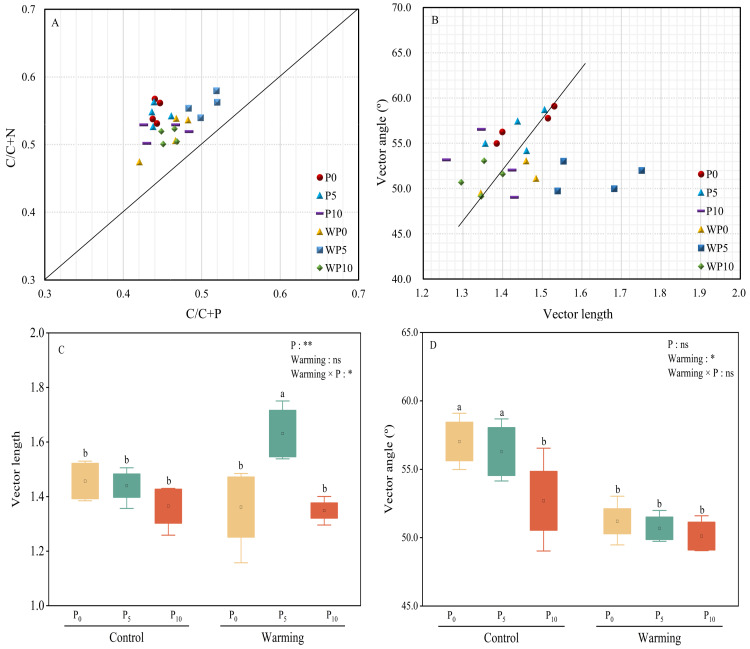
Scatter plots of soil enzyme stoichiometry of relative proportions of C/C+P to C/C+N (A). Regression analysis of vector length and vector angle (B). Warming, P addition, and their interaction on vector length (C) and vector angle (D). The results of two-way split-plot ANOVAs for the treatments are shown in the figures (*P < 0.05, ** *p* < 0.01, *** *p* < 0.001, and ns indicate no significance). Lowercase letters above the multi-factor group boxplots show significant differences among the treatments by Duncan’s test at *p* < 0.05. Error bars show means ± SE (*n* = 4).

All the treatments were over the 1:1 line (Fig. 5A), which showed that microbial nutrients were severely phosphorus limited. Vector lengths of P_0_ and WP_5_ were relatively greater than other treatments. Warming and P addition decreased vector lengths (Figs. 5B and 5C). Warming × P addition increased vector length by 11.64% at WP_5_ and decreased vector length by 7.53% at WP_10_. All the vector angles were greater than 45° , and vector angles were reduced by warming, P addition, and their interaction (Fig. 5D).

### Relationships between soil properties and soil extracellular enzyme activities

**Figure 6:**
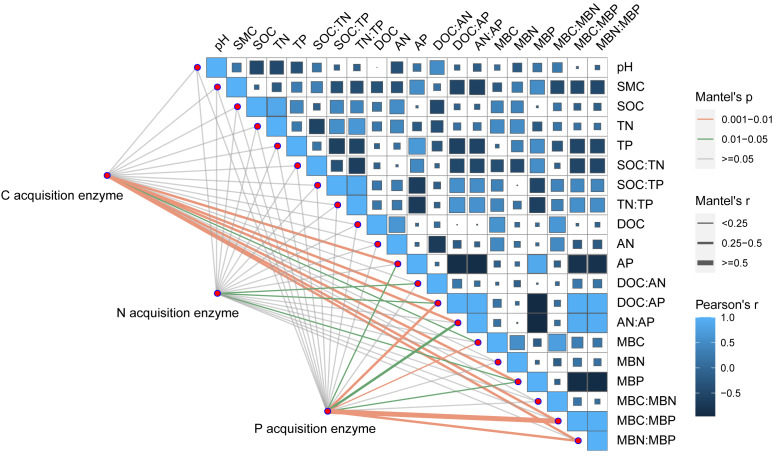
Correlation analysis on soil properties, soil extracellular enzyme activities, and stoichiometries. SOC, soil organic carbon; TN, soil total nitrogen; TP, soil total phosphorus; SMC, soil moisture content; DOC, dissolved organic carbon; AN, soil ammonium nitrogen (NH_4_^+^-N) and nitrate nitrogen (NO_3_^-^-N); AP, soil available phosphorus; MBC, microbial biomass carbon; MBN, microbial biomass nitrogen; MBP, microbial biomass phosphorus; C acquisition enzyme, *β*-1,4-glucosidase. N acquisition enzyme, leucine aminopeptidase and *β*-1,4-N-acetylglucosaminidase. P acquisition enzyme, alkaline phosphatase.

**Figure 7:**
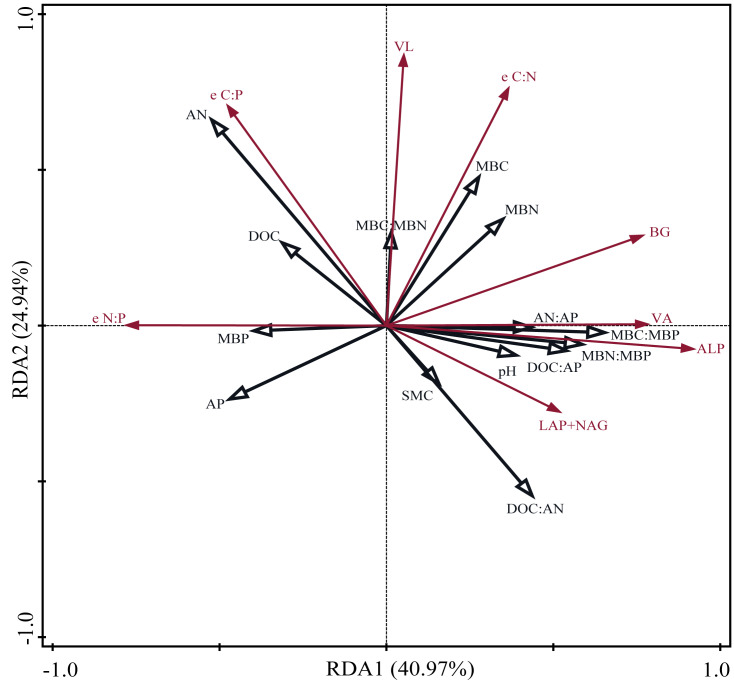
Redundancy analysis (RDA) on soil properties, soil extracellular enzyme activities and stoichiometries, vector length, and vector angle. SMC, soil moisture content; DOC, dissolved organic carbon; AN, soil ammonium nitrogen (NH_4_^+^-N) and nitrate nitrogen (NO_3_^-^-N); AP, soil available phosphorus; MBC, microbial biomass carbon; MBN, microbial biomass nitrogen; MBP, microbial biomass phosphorus; BG, *β*-1,4-glucosidase; LAP, leucine aminopeptidase; NAG, *β*-1,4-N-acetylglucosaminidase; ALP, alkaline phosphatase; e C:N, e C:P, and e N:P represent ln (BG): ln (LAP + NAG), ln (BG): ln (ALP) and ln(LAP +NAG): ln (ALP), respectively. VL, vector length. VA, vector angle.

Pearson correlation analysis showed a significant relationship between AP and DOC: AP, AN: AP, MBN: MBP, and MBC: MBP. Soil moisture content was significantly related to DOC: AP and AN: AP. The Mantel correlation analysis showed that soil total nutrients and stoichiometries had no significant effects on soil extracellular enzyme activities and stoichiometries. However, a positive link between soil C and P acquisition enzyme activities and soil available nutrients, microbial biomass, and their stoichiometry was observed. Soil N- acquiring enzyme activity was closely related to DOC: AN, DOC: AP, and MBP. (Fig. 6). The connection between soil available nutrients, soil extracellular enzyme activities and stoichiometries, and vectors were then examined (Fig. 7). The results revealed that the important factors affecting soil C-, N-, and P-acquiring activities and stoichiometries were AN, MBC: MBP, and MBN: MBP. There was a significant positive correlation between AN and soil e C: P; Soil e N: P was positively correlated with AP; MBC was positively correlated with soil e C: N; Vector length was positively correlated with AN, while vector angle was significantly negatively correlated with AP (Fig. 7).

## Discussion

### Short-term warming

Supporting our hypotheses, soil C-, N-, and P-acquiring enzymes were decreased by warming (Fig. 3). Warming increased soil available nitrogen contents (ammonium nitrogen and nitrate nitrogen, Table 1) and decreased microbial biomass C and N (Fig. 2), but there were no significant effects on the soil total nutrients and stoichiometries (Table 1). There was a significant positive correlation between soil extracellular enzyme activities (EEAs) and soil available nutrients, microbial biomass, and their stoichiometry (Figs. 6 and 7). These results indicated that increased temperature affected the secretion of soil enzymes by altering the available nutrients. This aligns with the results of previous studies (Cui et al., 2021; Li et al., 2022b). Further research on the soil microbial communities of desert steppes is required to better understand the exact microbiological mechanisms by which microbial biomass control soil extracellular enzyme activity.

Soil moisture content (SMC) may also affect soil extracellular enzyme activity (Allison & Treseder, 2008; Li et al., 2022a). Warming considerably decreased SMC in our study (Table 1), but had no significant impact on soil C-, N-, and P-acquiring activities (Figs. 6 and 7). It is suggested that soil microorganisms may adapt to dry, infertile soil conditions by adjusting soil extracellular enzyme activity in response to their demands and the surrounding environment (Wang et al., 2017). In our study, warming raised ln (BG): ln (ALP) and ln (LAP+NAG): ln (ALP), and decreased ln (BG): ln (LAP+NAG) (Fig. 3), vector length, and vector angle (Fig. 5). This is likely because warming reduced the soil microbial biomass C and N, and consequently influenced the secretion of C- and N-acquiring enzymes to adapt to environmental changes (Turner & Wright, 2014).

Seasons also have a significant effect on soil extracellular enzyme activity (Machmuller et al., 2016), which may indicate variations in substrate accessibility and environmental circumstances across different seasons. A long-term warming experiment is needed to observe the larger impact of seasonality on soil extracellular enzyme activity. In this study, short-term warming changed soil microbial activities and stoichiometries in desert steppe.

### Phosphorus addition

Soil phosphatases typically showed higher activity in P-limited ecosystems, and soil C- and N-acquiring enzyme activities reflect microbial demand for energy and nutrients (Turner & Wright, 2014). In our study, P addition decreased soil extracellular enzyme activities (Fig. 3), which had a great impact on P acquisition enzyme activity (Marklein & Houlton, 2012; Shi et al., 2021). P addition significantly promoted soil total P and available P contents while concurrently reducing DOC and AN (Table 1). P addition significantly increased MBP and decreased MBC and MBN (Fig. 2), suggesting that soil microorganisms may preferentially fix P because low inorganic P availability induces rapid microbial P uptake (Bünemann et al., 2012). Our correlation analysis demonstrated a negative correlation between soil C-, P- acquiring enzyme activity and available P, MBP (Fig. 6). This relationship is most likely explained by P addition increasing plant growth and nutrient uptake while reducing the number of nutrients available for the synthesis of soil enzymes (Wang, Wang & Liu, 2008). These results are contrary to the results of some previously published research, which can likely be explained by differing factors such as the rate of P addition, type of ecosystem, and study site (Colvan, Syers & O’Donnell, 2001; Tian et al., 2016; Wang et al., 2020b).

In terms of soil extracellular enzyme stoichiometry, P addition decreased ln (BG): ln (LAP+NAG), and increased ln (BG): ln (ALP) and ln (LAP+NAG): ln (ALP) (Fig. 3). This finding illustrates that P addition decreased microbial demand for carbon and soil microbial demand for alkaline phosphatase production costs in phosphorus-limited soils. This may also impact nutrient cycling, plant productivity, and microbial community composition (Peñuelas et al., 2013; Luo et al., 2019). P addition decreased vector length and vector angle (Fig. 5), demonstrating that P addition can alleviate both the soil microbial carbon and phosphorus limitation (Wang et al., 2022). This result confirmed our hypothesis that soil microbial nutrient limitation of the desert steppe is closely related to soil available P.

### The interaction effects of short-term warming and P addition

Warming × P addition significantly reduced soil C-, N- and P-acquiring enzyme activities (Fig. 3). As for C-acquiring enzyme activity, WP_5_ was higher than WP_10_, which may be related to the significant increase in soil DOC and AN. This, in turn, may have caused the low level of P_5_ addition rate to secrete more C and N acquisition enzymes at higher temperatures (Fig. 2). At WP_10_, there was less soil available C and N, and fewer C- acquiring enzymes were secreted by the microorganisms. WP_10_ was consistently higher than WP_5_ throughout the changes in N-, and P-acquiring enzyme activities, suggesting that the amount of available phosphorus may be a vital factor (Table 1). P-acquiring enzymes were most significantly impacted by warming × P addition (Fig. 3), indicating a negative feedback relationship between high available phosphorus content and soil extracellular enzyme activities (Allison et al., 2007). The positive correlation between BG, LAP+NAG, and ALP indicates that the microbial acquisition to C, N, and P were altered due to the changes in soil substrate availability (Sinsabaugh, Hill & Shah, 2009). Warming × P addition resulted in a reduction of ln (BG): ln (ALP) and ln (LAP+NAG): ln (ALP), an increase in ln (BG): ln (LAP+NAG) at WP_5_, and a decline in ln (BG): ln (LAP+NAG) at WP_10_ (Fig. 3). The primary factors that influenced soil extracellular enzyme activities and stoichiometries were AN, MBC: MBP, and MBN: MBP. These results show that the interaction of warming and P addition had an impact on soil physicochemical properties, which altered the stoichiometry of soil extracellular enzymes (Zheng et al., 2015; Zhang et al., 2018).

Additionally, the natural log ratio of soil C-, N-, P- acquiring enzymes in this study was 1.2:1:1.3 (Table S1), which differs from the global mean scale of 1:1:1 (Sinsabaugh, Hill & Shah, 2009), demonstrating that the desert steppe in the study region was largely C and P limited. This is partially consistent with the findings of a previous study demonstrating that a desert steppe was severely P-limited in the temperate grassland of northern China (Peng & Wang, 2016). The ratio of soil C: N: P acquisition enzymes adjusted to 1.3:1:1.2 at WP_5_. A low phosphorus addition rate caused both soil microbial carbon and phosphorus limitation in the region, while the addition of P_10_ alleviated it (Table S1). Previous research revealed that adding P may boost soil microbial nitrogen demand, and lead to an increase in N-acquiring enzyme activity (Wang et al., 2020b). However, this did not occur in our study, and this finding may need to be confirmed by further research. Our vector analysis revealed that relative carbon limitation increased at WP_5_ and decreased at WP_10_. A higher level of P addition rate (P_10_) was beneficial to alleviating P limitation in the region. Warming × P addition changed the soil available nutrients and their stoichiometry, while soil nutrient stoichiometry influenced microbial C, N, and P metabolism by regulating soil elemental balance (Cui et al., 2019b). In this process, the secretion of soil microbial enzymes in P-deficient areas of the desert steppe was stimulated to adapt to nutrient limitation (Schimel, Balser & Wallenstein, 2007). According to our findings, the key influencing factors of microbial nutrient restriction in desert steppe are soil available nutrients, microbial biomass, and stoichiometries.

## Conclusions

In summary, warming did not affect soil available P but altered soil ammonium nitrogen and nitrate nitrogen, microbial biomass C, and N. P addition significantly raised soil available phosphorus contents, which had positive effects on the activities of soil C-, N-, and P-acquiring enzymes. For the different rates of P addition, P_10_ was effective in alleviating the relative carbon and phosphorus limitation compared to P_5_. Warming and P addition altered soil available nutrients, microbial biomass, and their stoichiometry while decreasing microbial C and P demand. The primary factors that influenced the soil extracellular enzyme activities and stoichiometries were AN, MBC: MBP, and MBN: MBP. In conclusion, short-term warming, P addition, and their interaction significantly affected soil extracellular enzyme activities and stoichiometries, which in turn changed microbial resource acquisition techniques in the desert steppe. These findings may provide useful insights into the adaptation to climate warming in desert steppe. To confirm the potential role that soil available phosphorus might play in actively adapting to global warming and microbial nutrient limitations, further study on long-term warming and soil microbial community assessment is necessary.

##  Supplemental Information

10.7717/peerj.16227/supp-1 Supplemental Information 1 Raw data

10.7717/peerj.16227/supp-2 Supplemental Information 2 Open-top chambers (OTCs) and control treatments arrangements at the study sites A randomized split-plot design with two temperature levels (Control, CK; Warming, W) was used as the main plots, and three P addition levels (0 g m^−2^ yr^−1^, 5 g m^−2^ yr^−1^ , and 10 g m^−2^ yr^−1^) were used as the subplots. The main plots were separated from each other by 3 m.

10.7717/peerj.16227/supp-3 Supplemental Information 3 Mean annual temperature and mean annual precipitation in the study site from 1980 to 2021

10.7717/peerj.16227/supp-4 Supplemental Information 4 Ratios of ln-transformed C, N, and P acquisition enzymes for the different treatments BG, *β*-1,4-glucosidase (nmol^−1^ g^−1^ h^−1^). NAG, *β*-1,4-Nacetylglucosaminidase (nmol^−1^ g^−1^ h^−1^). LAP, leucine aminopeptidase (nmol g h). ALP, alkaline phosphatase (nmol^−1^ g^−1^ h^−1^).

## Additional Information and Declarations

### Data Availability

The following information was supplied regarding data availability: The raw measurements are available in the Supplemental Files.
