# Supplementary figures and images for: Response of soil extracellular enzyme activity and stoichiometry to short-term warming and phosphorus addition in desert steppe
Source: PeerJ. 2023 Oct 19;11:e16227. doi: 10.7717/peerj.16227 (PMC10590576; doi:10.7717/peerj.16227)

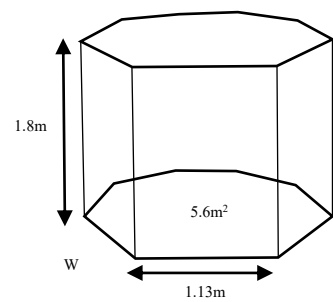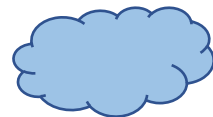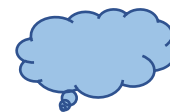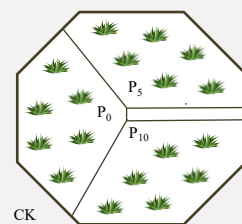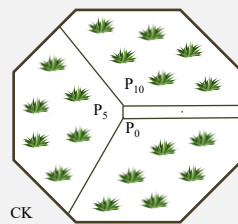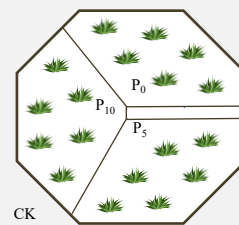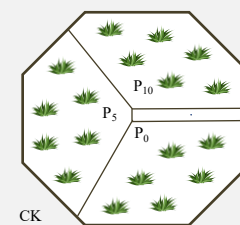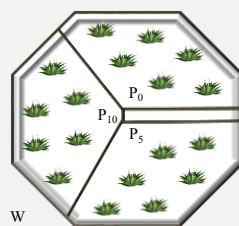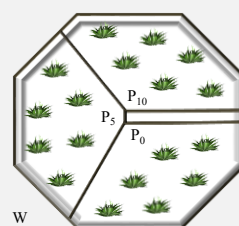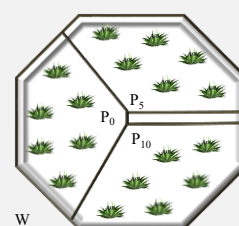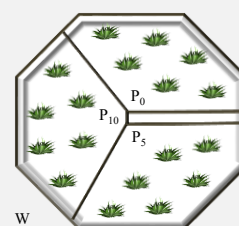

Supplement: Supplemental Information 2 — A randomized split-plot design with two temperature levels (Control, CK; Warming, W) was used as the main plots, and three P addition levels (0 g m−2 yr−1, 5 g m−2 yr−1 , and 10 g m−2 yr−1) were used as the subplots. The main plots were separated from each other by 3 m. [file peerj-11-16227-s002.pdf]

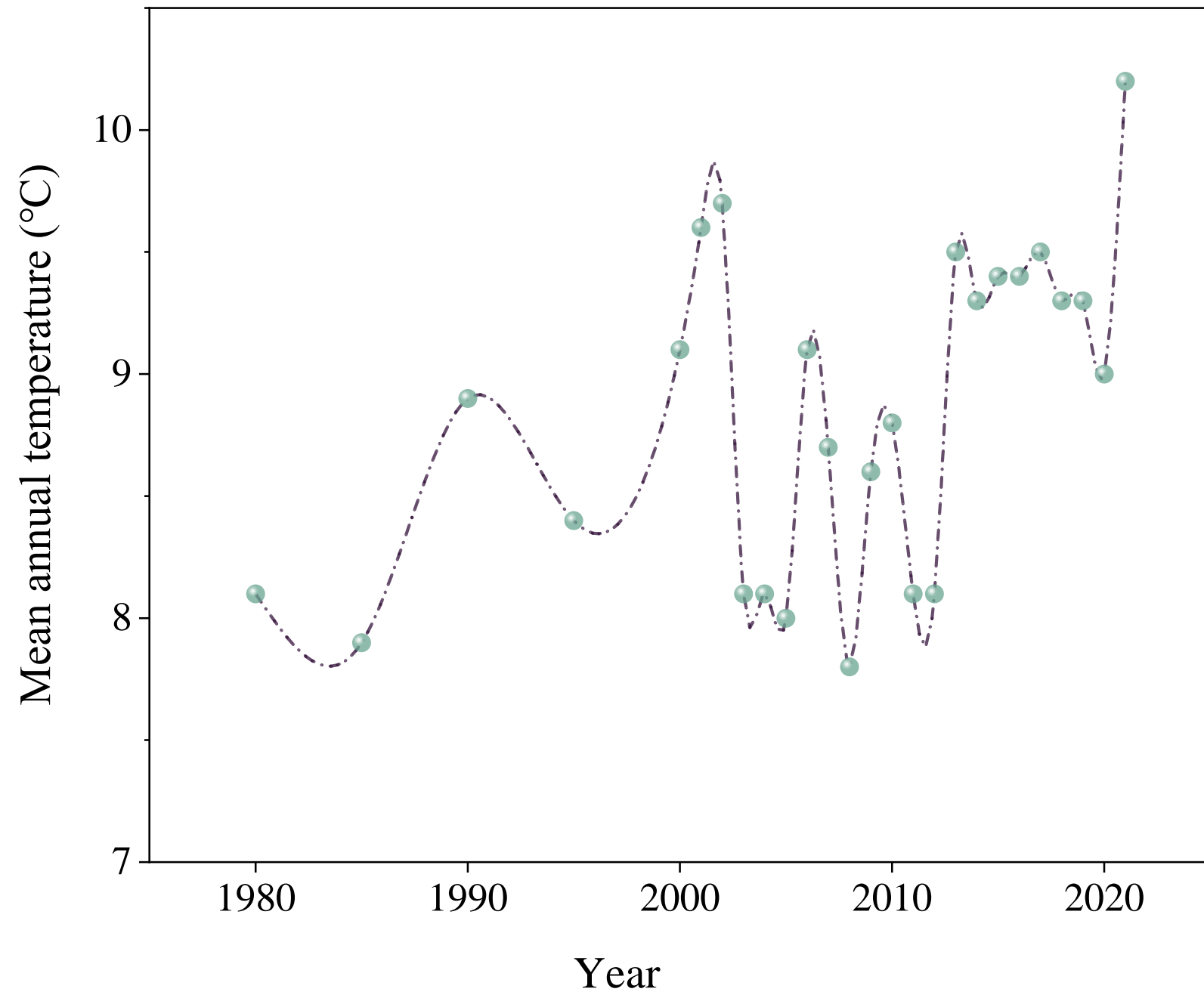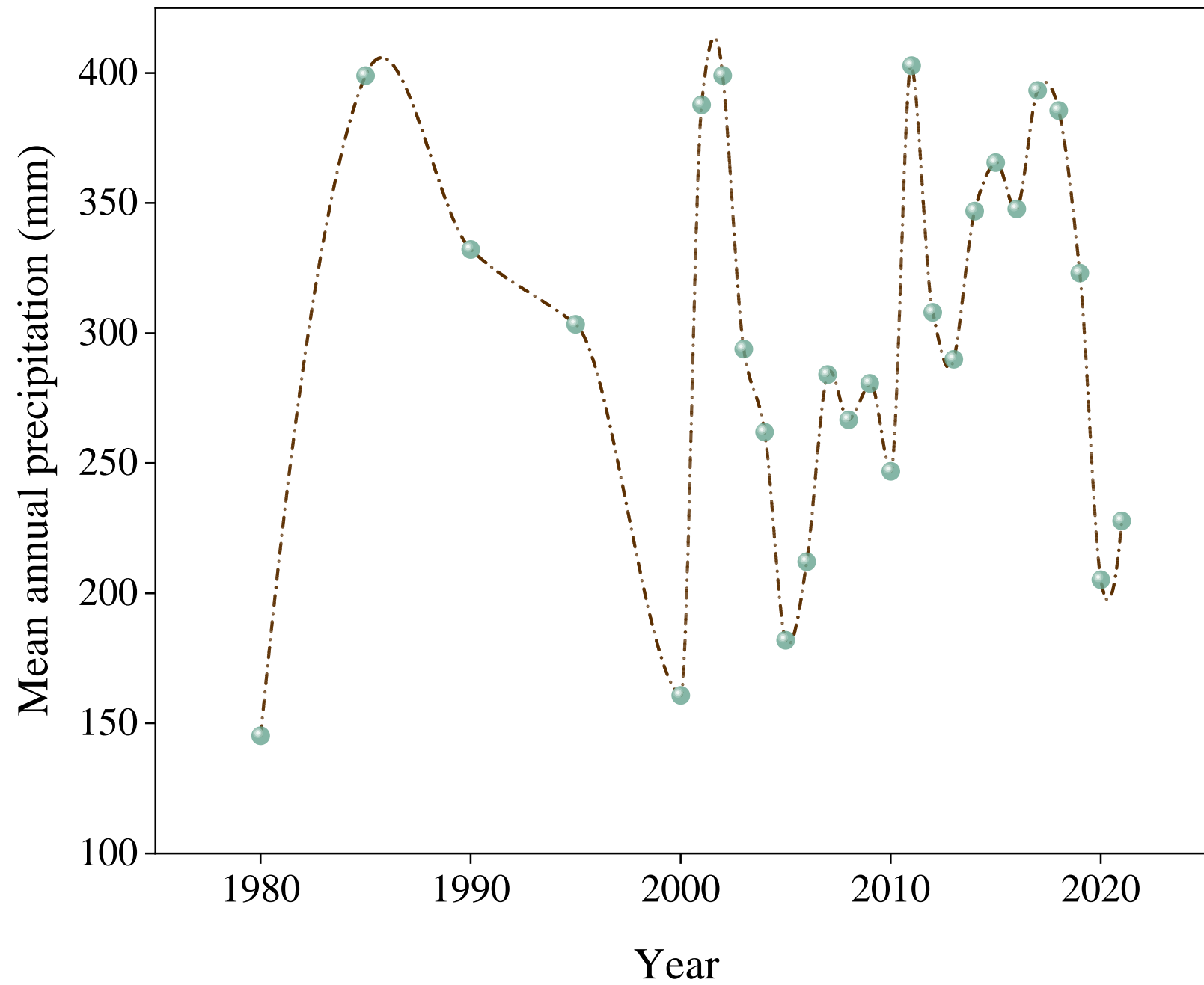

Supplement: Supplemental Information 3 [file peerj-11-16227-s003.pdf]
