# Supplementary material for: Response of soil extracellular enzyme activity and stoichiometry to short-term warming and phosphorus addition in desert steppe
Source: PeerJ. 2023 Oct 19;11:e16227. doi: 10.7717/peerj.16227 (PMC10590576; doi:10.7717/peerj.16227)
Supplement: Supplemental Information 4 — BG, β-1,4-glucosidase (nmol−1 g−1 h−1). NAG, β-1,4-Nacetylglucosaminidase (nmol−1 g−1 h−1). LAP, leucine aminopeptidase (nmol g h). ALP, alkaline phosphatase (nmol−1 g−1 h−1). [file peerj-11-16227-s004.docx]

| Treatment | | Soil ln(BG):ln(LAP+NAG):ln(ALP) ratios |
| --- | --- | --- |
| Control | P_0_ | 1.2:1:1.5 |
|  | P_5_ | 1.2:1:1.5 |
|  | P_10_ | 1.1:1:1.3 |
| Warming | P_0_ | 1.1:1:1.2 |
|  | P_5_ | 1.3:1:1.2 |
|  | P_10_ | 1:1:1.2 |
